# Risk assessment of shoulder dystocia via the difference between transverse abdominal and biparietal diameters: A retrospective observational cohort study

**DOI:** 10.1371/journal.pone.0247077

**Published:** 2021-02-12

**Authors:** Satoshi Shinohara, Yasuhiko Okuda, Shuji Hirata

**Affiliations:** Department of Obstetrics and Gynecology, Faculty of Medicine, University of Yamanashi, Chuo, Yamanashi, Japan; University of Insubria, ITALY

## Abstract

Shoulder dystocia is defined as vaginal cephalic delivery that requires additional obstetric maneuvers to deliver the fetus after the head has been delivered and gentle traction has failed. A bigger difference between the transverse abdominal diameter (TAD) (abdominal circumference [AC]/π) and biparietal diameter (BPD) (TAD-BPD) has been reported as a risk factor for shoulder dystocia in different countries; however, it remains unclear if this relationship is relevant in Japan. This study aimed to clarify the association between TAD-BPD and shoulder dystocia after adjusting for potential confounding factors in a Japanese cohort. We retrospectively examined 1,866 Japanese women who delivered vaginally between 37+0 and 41+6 weeks of gestation at the University of Yamanashi Hospital between June 2012 and November 2018. The cutoff value of TAD-BPD associated with shoulder dystocia and the association between TAD-BPD and shoulder dystocia were evaluated. The mean maternal age was 32.5±5.3 years; the patients included 1,053 nulliparous women (57.5%), 915 male infants (49.0%), 154 women with gestational diabetes mellitus (GDM) (8.3%), and 5 infants with macrosomia (0.3%). The mean TAD-BPD was 9.03±4.7 mm. The overall incidence of shoulder dystocia was 2.4% (44/1866). The cutoff value to predict shoulder dystocia was 12.0 mm (sensitivity, 61.4%; specificity, 73.8%; likelihood ratio, 2.34; positive predictive value, 5.4%; negative predictive value, 98.8%). We then used a multivariable logistic regression analysis to examine the association between TAD-BPD and shoulder dystocia while controlling for the potential confounding factors. In multivariate analyses, TAD-BPD ≥12.0 mm (adjusted odds ratio [OR], 4.39; 95% confidence interval [CI], 2.35–8.18) and GDM (adjusted OR, 3.59; 95% CI, 1.71–7.52) were associated with shoulder dystocia. Although TAD-BPD appears to be a relevant risk factor for shoulder dystocia, sonographic fetal anthropometric measures do not appear to be useful in screening for shoulder dystocia due to a low positive predictive value.

## Introduction

Shoulder dystocia is defined as vaginal cephalic delivery that requires additional obstetric maneuvers to deliver the fetus after the head has been delivered and gentle traction has failed [1, 2]. Shoulder dystocia, which is associated with increased rates of maternal and fetal morbidities, occurs in approximately 0.2% to 3.0% of vaginal births [2]. For the mother, shoulder dystocia increases the risk of postpartum hemorrhage as well as third- and fourth-degree perineal lacerations [3, 4]. Similarly, in neonates, shoulder dystocia increases the risk of asphyxia, permanent Erb’s palsy, and fetal fractures [5]. Although the incidence of shoulder dystocia has been increasing in recent years, predicting the risk of shoulder dystocia in the clinical settings is difficult. Therefore, a complete understanding of the risk factors associated with shoulder dystocia and the development of appropriate treatment strategies are essential. Shoulder dystocia may be attributed to several factors, such as macrosomia, maternal obesity, gestational diabetes mellitus (GDM), induction of labor, prolonged first and second stages of labor, and assisted vaginal delivery [6]. The difference between the transverse abdominal diameter (TAD) (abdominal circumference [AC]/π) and biparietal diameter (BPD) (TAD-BPD), which is calculated easily using antepartum sonographic fetal biometry, has been reported to be useful for assessing the risk of shoulder dystocia [8, 9]. Previous studies have reported that large TAD-BPD increases the risk of shoulder dystocia [7–9]. However, to the best of our knowledge, no studies have assessed the relationship between increased TAD-BPD and shoulder dystocia in the Japanese population. Furthermore, it remains unclear whether this relationship is relevant to the Japanese population because the maternal physique differs according to race and ethnicity [10]. In particular, pregnant women in Japan are physically shorter and thinner than those in Western countries [10]. Prenatal check-ups in Japan are conducted weekly during 36–39 weeks of gestation and twice weekly after 40 weeks [11]. Japanese obstetricians calculate BPD and AC at each prenatal check-up to estimate the fetal weight using the Shinozuka formula, which is as follows: EFW (g) = 1.07 × BPD^3^ + 3.00 × 10^−1^AC^2^ × FL, where BPD is the biparietal diameter (cm), AC is the abdominal circumference (cm), and FL is the femur length (cm) [12]. Consequently, in term pregnancies, sonographic measurements of BPD, AC, and TAD within 7 days of delivery are readily available to obstetricians in the clinical settings. A better understanding of the relationship between TAD-BPD before delivery and shoulder dystocia will provide clinically useful information for perinatal management in Japan. The aim of this retrospective study was to evaluate the association between fetal TAD-BPD, as measured using ultrasonography prior to delivery in term pregnancies, and shoulder dystocia in a Japanese cohort.

## Materials and methods

### Study design

We conducted this retrospective observational cohort study at the University of Yamanashi Hospital between June 2012 and November 2018. The study included women with singleton pregnancies at 37+0 to 41+6 weeks of gestation in whom sonographic measurements of BPD and TAD within 7 days of vaginal delivery were available. Women with estimated fetal weight (EFW) < 2,500 g within 7 days of delivery (n = 114) were excluded, except for women who may have fetal microcephaly and to consider in a more general population. Women with missing data (n = 16) were also excluded. Indications for operative vaginal delivery to shorten and reduce the effects of the second stage of labor included a non-reassuring fetal status and lack of continuing labor progress for at least 2 hours irrespective of nulliparous or multiparous status [11]. The study protocol was reviewed and approved by the Human Subjects Review Committee of the University of Yamanashi Hospital, and the requirement for informed consent from patients was waived due to the retrospective study design. Nevertheless, patients were provided the opportunity to refuse the use of their data through the hospital’s website. All procedures were performed in accordance with the 1964 Helsinki Declaration and its later amendments.

### Data collection

We collected the obstetric data from the medical records. Gestational age was determined based on the last reported menstrual period and was confirmed by the crown-rump length on the first-trimester sonogram. We recorded the following data: mother’s age at delivery, fetal sex, GDM status, parity, gestational age at delivery, maternal stature, and pre-pregnancy weight. Additionally, we included details of induction of labor and macrosomia, which are potential confounding factors that have been reported to be risk factors for shoulder dystocia [6]. The dose and type of uterine contraction agent (oxytocin or prostaglandin) were determined by the treating obstetrician during induction or augmentation of labor according to the guidelines for obstetrical practice in Japan [11]. Our facility does not perform delivery with epidural anesthesia. Fetal BPD was measured from the outer edge of the proximal calvarium to the inner edge of the distal calvarium (outer-inner) at the level of the 3^rd^ ventricle and thalami [13, 14]. AC was measured in the transverse view of the abdomen at the level of the junction of the umbilical vein and left portal vein [7]. The presence of shoulder dystocia was defined according to a previous study and the American College of Obstetricians and Gynecologists (ACOG) practice guidelines as “the requirement of additional obstetric maneuvers to release the shoulders after gentle downward traction has failed” [15]. The diagnosis of GDM was established if there was at least one abnormal plasma glucose value (≥ 92, 180, and 153 mg/dL as fasting, 1-h, and 2-h plasma glucose concentrations, respectively) after a 75-g oral glucose tolerance test [11]. Advanced maternal age was defined as a maternal age of over 35 years [11]. Body mass index (BMI) before pregnancy was calculated according to the World Health Organization standards, and the patients were classified as obese (≥ 25.0 kg/m^2^) or non-obese (< 25.0 kg/m^2^) according to the Japan Society of Obstetrics and Gynecology Guidelines for Obstetrical Practice 2014 [11]. Macrosomia was defined as birth weight ≥ 4,000 g [16].

### Statistical analyses

We have compiled a list of collected perinatal data to identify patients easily. Mann–Whitney U and chi-squared tests were first used to determine the potential confounding factors for shoulder dystocia. Subsequently, a receiver-operating characteristic (ROC) curve was used to determine the optimal TAD-BPD cutoff value for predicting shoulder dystocia. We used the Youden index, which describes the maximum vertical distance between the ROC curve and the diagonal or chance line, to define the optimal cutoff value [17]. Finally, a multiple logistic regression model was used to identify the variables significantly associated with shoulder dystocia. The logistic regression models were adjusted using the TAD-BPD cutoff value, GDM status, sex of the infant, advanced maternal age, obesity, instrumental delivery, and estimated fetal weight (EFW), which are potential confounding factors that have been reported to be risk factors for shoulder dystocia in the previous study [6]. All analyses were performed using Bell Curve for Excel (Social Survey Research Information Co., Ltd., Tokyo, Japan), and the significance level was set at P < 0.05.

## Results

**Fig 1:**
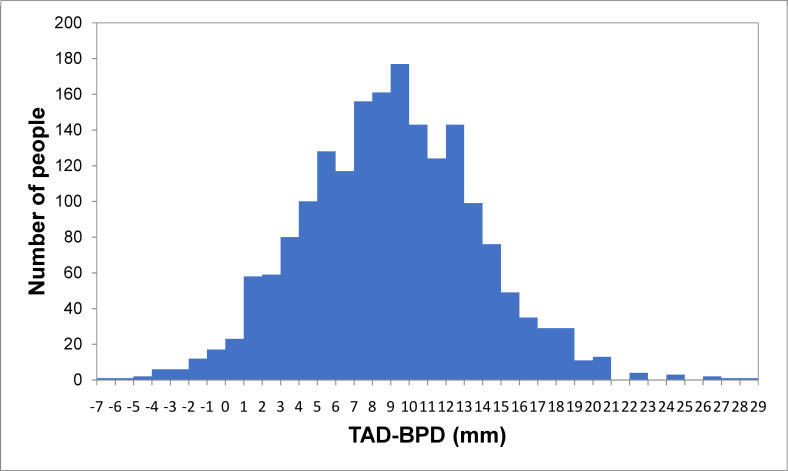
Histogram of the number of patients. TAD, transverse abdominal diameter; BPD, biparietal diameter.

**Fig 2:**
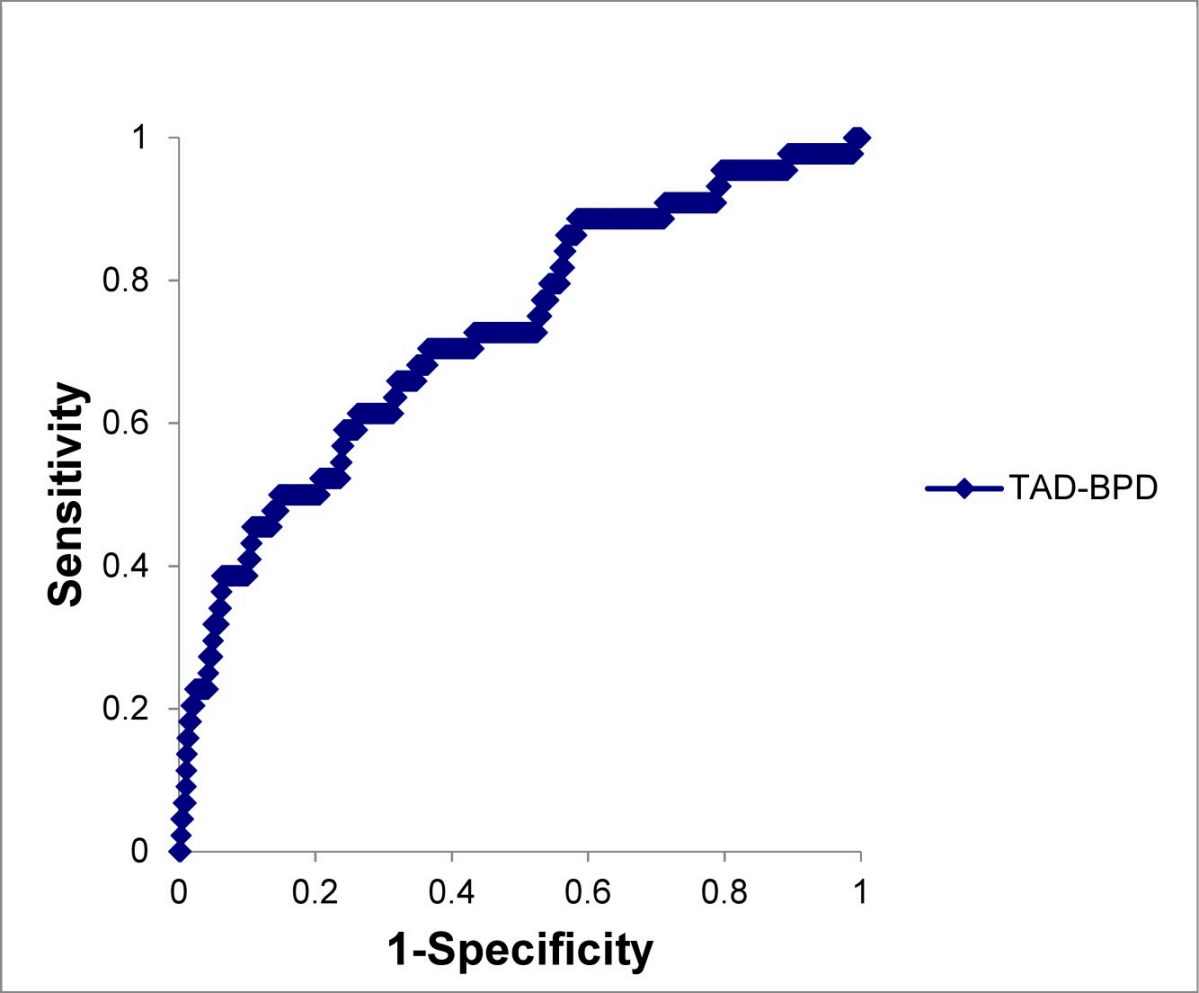
Receiver-operating curve analysis of TAD-BPD for predicting shoulder dystocia in a cohort of 1,866 women. TAD, transverse abdominal diameter; BPD, biparietal diameter.

A total of 1,866 women were included in this study. The overall mean maternal age was 32.5 ± 5.3 years, and the mean maternal pre-pregnancy BMI was 21.3 ± 3.6 kg/m^2^. The patients included 1,053 nulliparous women (57.5%), 915 male infants (49.0%), 154 women with GDM (8.3%), and 5 infants with macrosomia (0.3%). The mean TAD-BPD was 9.03 ± 4.7 mm (Fig 1). The overall incidence of shoulder dystocia was 2.4% (44/1866). Based on ROC analysis, a TAD-BPD cutoff of 12.0 mm would allow for the maximum number of participants to be correctly classified based on the presence of shoulder dystocia. A cutoff of 12.0 mm provided sensitivity of 61.4%, specificity of 73.8%, likelihood ratio of 2.34, positive predictive value of 5.4%, and negative predictive value of 98.8% (area under the curve: 0.74) (Fig 2). Table 1 summarizes the clinical characteristics of the enrolled women. Pre-pregnancy BMI, birth weight, shoulder dystocia, and intrapartum hemorrhage were significantly higher, and nulliparity was lower in the group with TAD-BPD ≥ 12.0 mm than in the group with TAD-BPD < 12.0 mm (Table 1).

**Table 1:** Baseline characteristics of the study population.

Characteristic	TAD−BPD < 12.0 mm n = 1,362	TAD−BPD ≥ 12.0 mm n = 504	P-value
Maternal age, years	32.4 ± 5.2	32.5 ± 5.3	0.74
Nulliparity	803 (58.9)	250 (49.6)	<0.001
Pre-pregnancy BMI, kg/m^2^	21.1 ± 3.5	21.8 ± 3.6	<0.001
Birth weight, g	3,038 ± 325	3,319 ± 368	<0.001
Shoulder dystocia	17 (1.3)	27 (5.4)	<0.001
UA pH	7.32 ± 0.07	7.31 ± 0.06	0.92
Amount of bleeding at delivery, g	423 ± 314	467 ± 355	0.01
Instrumental delivery	122 (8.9)	35 (6.9)	0.16

Values are presented as average ± standard deviation or as numbers (%).

biparietal diameter (BPD); BMI, body mass index; transverse abdominal diameter (TAD); UA, umbilical arterial.

In the multivariate analyses, TAD-BPD ≥ 12.0 mm (adjusted odds ratio [OR], 4.45; 95% confidence interval [CI], 2.38–8.33) and GDM (adjusted OR, 3.71; 95% CI,1.72–8.02) were associated with shoulder dystocia (Table 2).

**Table 2:** Factors associated with shoulder dystocia.

Variables	Shoulder dystocia (n = 44)	Non-shoulder dystocia (n = 1,822)	Crude		Adjusted	
			OR	95% CI	OR	95% CI
TAD-BPD ≥ 12.0 mm						
No	17	1345	1.0	Reference	1.0	Reference
Yes	27	477	3.42	1.66–7.08	4.46	2.38–8.33
GDM						
No	34	1678	1.0	Reference	1.0	Reference
Yes	10	144	3.42	1.66–7.08	3.71	1.72–8.05
Male infant						
No	21	930	1.0	Reference	1.0	Reference
Yes	23	892	1.14	0.63–2.08	1.15	0.63–2.12
Maternal age, years						
< 35	27	1144	1.0	Reference	1.0	Reference
≥ 35	17	678	1.06	0.57–1.96	0.94	0.50–1.77
Obesity						
No	38	1613	1.0	Reference	1.0	Reference
Yes	6	209	1.22	0.51–2.92	0.85	0.34–2.14
Instrumental delivery						
No	40	1669	1.0	Reference	1.0	Reference
Yes	4	153	1/09	0.39–3.08	1.19	0.41–3.46
EFW, g						
< 4,000	43	1818	1.0	Reference	1.0	Reference
≥ 4,000	1	4	10.6	1.16–96.6	5.25	0.56–49.5

BPD, biparietal diameter; EFW, estimated fetal weight; GDM, gestational diabetes mellitus; TAD, transverse abdominal diameter.

## Discussion

In this study on Japanese women, the following two important clinical findings were observed: (1) increased TAD-BPD was an independent risk factor of shoulder dystocia, and (2) TAD-BPD was not applicable as a screening tool for shoulder dystocia because the positive predictive value was very low (5.4%).

Burkhardt et al. reported that maternal diabetes is an independent risk factor for shoulder dystocia. Additionally, the typical disproportion in these fetuses in favor of the trunk has been hypothesized to be important in fetal macrosomia [7]. Birthweight has been generally accepted to be directly related to insulin sensitivity, therefore indicating that maternal glucose metabolism may play an important role in fetal growth. Glucose crosses the placenta in cases of impaired maternal glycemic control and high maternal serum glucose levels; however, maternal insulin does not cross the placenta. Consequently, during the second trimester, the fetal pancreas, which is now capable of secreting insulin, can now respond to hyperglycemia and secretes insulin autonomously irrespective of glucose stimulation. Since insulin is a major anabolic hormone and glucose is a major anabolic fuel, the combination of hyperinsulinemia and hyperglycemia leads to an increase in fat and protein stores by the fetus [18–20]. Insulin sensitivity has been reported to vary between organs: the liver, muscles, and subcutaneous fat have high insulin sensitivity, while the brain and bones have low insulin sensitivity [21]. Fetal disproportion may be attributed to differences in insulin sensitivity between these organs. In this study, GDM was found to be an independent risk factor for shoulder dystocia. Although the lack of universally accepted criteria makes the definition of diagnosis and prognosis of GDM difficult, early diagnosis and glucose blood level control may improve maternal and fetal short- and long-term outcomes [22]. Nutritional interventions, exercise, and medical treatment such as insulin therapy have been reported to be effective for glucose blood level control in women with women [22–24]. Recently, some studies reported that myo-inositol and vitamin D seem to be effective and safe for the prevention and treatment of GDM [22–24]. Therefore, to reduce the onset of shoulder dystocia, continuation of conventional treatments for GDM and new research for improvement in GDM are needed in the future. Furthermore, in some pregnant women who were not diagnosed with GDM but developed shoulder dystocia, their glucose metabolism might have been similar to that of patients with GDM.

Although previous studies had slightly different definitions of shoulder dystocia, TAD-BPD cutoffs, and study populations, several studies have reported an association between TAD-BPD values and shoulder dystocia [7–9]. The findings of the current study are consistent with previous studies demonstrating that TAD-BPD has poor predictive ability for shoulder dystocia (Table 3) [7, 9]. However, our study analyzed more risk factors for shoulder dystocia in a relatively larger sample size in Japanese women [7–9]. Although it is well-known that maternal physique and glucose metabolism vary according to race and ethnicity, to the best of our knowledge, this is the first study to investigate the association between TAD-BPD and shoulder dystocia in Japanese women [10].

**Table 3:** Factors associated with shoulder dystocia.

Reference	Study period	Cutoff, mm	Sensitivity	Specificity	PPV	NPV
Burkhardt [7]	1995–2011	26.0	8.2	98.8	7.55	98.9
Miller RS [9]	2003–2005	26.0	44.4	90.0	25.0	96.0
Present study	2012–2018	12.0	61.4	73.8	5.40	98.8

PPV, positive predictive value; NPV, negative predictive value

Since the degree of fetal asymmetry, as evaluated by TAD-BPD, appears to be directly associated with the incidence of shoulder dystocia according to the current and previous studies, this factor alone is not a reliable predictor of shoulder dystocia due to its low positive predictive value. Previous reports have indicated that GDM is a major risk factor for shoulder dystocia, and our findings support this conclusion [6]. Therefore, we evaluated the association between TAD-BPD and shoulder dystocia in women with GDM. Although a multivariable analysis was not possible due to the small sample size, the ability of TAD-BPD to predict shoulder dystocia was examined. In the present study, based on a ROC analysis of 156 women with GDM, we found that a cutoff of 12.3 mm provided a sensitivity of 80.0%, specificity of 78.5%, likelihood ratio of 3.72, positive predictive value of 18.9%, and negative predictive value of 97.4% (area under the curve: 0.87). On the contrary, based on a ROC analysis of 1712 women with non-GDM, we found that a cutoff of 13.5 mm provided a sensitivity of 47.1%, specificity of 85.2%, likelihood ratio of 3.18, positive predictive value of 6.0%, and negative predictive value of 98.8% (area under the curve: 0.69). Therefore, when our study population were limited to women with GDM, TAD-BPD alone demonstrated relatively high predictive ability for shoulder dystocia. Obstetricians need to recognize that it is difficult to predict shoulder dystocia using ultrasound data. However, only for women with GDM, elective cesarean section may be considered if TAD-BPD is very high and the woman strongly desires an elective cesarean section. However, in such cases, it is necessary to explain the maternal complications associated with a cesarean section [25].

Furthermore, a multiple logistic regression model was used with TAD-BPD as a continuous variable. Subsequently, 1.20 (95% CI, 1.13–1.28; P < 0.001) for each 1-mm increase in the TAD-BPD. Additionally, TAD-BPD can be routinely determined without additional costs and therefore, might appear meaningful at first glance for obstetricians. However, considering that the incidence of shoulder dystocia ranges from 0.2% to 3.0% and the negative predictive value for TAD-BPD was 98.8% in this study, TAD-BPD might not be a useful and valuable screening method for shoulder dystocia. The positive and negative predictive values are affected by the prevalence; therefore, considering that our study was performed at a single center, the results should be interpreted with caution.

This study has certain limitations. First, this was a single-center study; therefore, it may be difficult to generalize our results to the general population. According to the sample size calculation method previously reported and widely used for logistic analyses, “ten events per variable” is a widely adopted minimal criterion for logistic regression analyses [26]. Based on this method, we needed at least 70 women with shoulder dystocia in this study. Since this study only included 44 women with shoulder dystocia, there is a possibility of insufficient power of the study in detecting a risk difference. Therefore, a large-scale, multicenter, prospective cohort study is required to confirm our results in the general population. Second, cases of prolonged second stage of labor and diabetes in pregnancy could not be included due to insufficient information from the electronic medical records; therefore, there is the possibility that unmeasured confounders may be associated with shoulder dystocia [6].

In conclusion, although TAD-BPD appears to be a relevant risk factor for shoulder dystocia, it has poor predictive ability for shoulder dystocia. Risk assessments of shoulder dystocia using fetal TAD-BPD may not lead to improved perinatal prognoses in the Japanese population.
